# Saúde e ecologia na Luta do Larzac, França, 1971-1981: relações entre corpo e território na formação de uma sensibilidade ecológica

**DOI:** 10.1590/S0104-59702025000100025

**Published:** 2025-05-23

**Authors:** Renata Palandri Sigolo, Sylvain Ferez

**Affiliations:** i Professora, Departamento de História/Universidade Federal de Santa Catarina. Florianópolis – SC – Brasil r.palandri@ufsc.br; ii Professor, Université de Montpellier. Montpellier – Hérault – França sylvain.ferez@umontpellier.fr

**Keywords:** Larzac (França, Movimento ecológico, Medicinas alternativas, Medicinas heterodoxas, Movimentos sociais, Larzac (France, Ecology movement, Alternative medicine, Unorthodox medicine, Social movements

## Abstract

O objetivo principal deste texto é analisar as relações entre práticas alternativas de saúde e meio ambiente na França, mais especificamente durante a Luta do Larzac, um movimento social ocorrido entre 1971 e 1981. Visando resistir à expropriação de terras agriculturáveis pelo Exército para a expansão de um campo militar, o movimento criou vários grupos de resistência. Entre eles, destacamos Larzac-Université e o primeiro curso oferecido pelo grupo, que foi o objeto de nossa análise: ocorrido em 1976 e denominado “Os produtos químicos, os medicamentos, a energia: uma ameaça à vida cotidiana?”, ele nos fornece pistas de compreensão da conexão entre saúde, meio ambiente e movimentos sociais.

## Abstract (en)

The main objective of this text is to analyze the relationships between alternative health practices and the environment in France, especially during the Battle of Larzac, a social movement that occurred between 1971 and 1981. In its bid to prevent the expropriation of agricultural land by the army for the expansion of a military camp, the movement created several resistance groups. We highlight Larzac-Université and the first course offered by the group, which is analyzed here. This course, held in 1976, called “Chemicals, medicines, energy: a threat to everyday life?”, sheds light on the connections between health, the environment, and social movements.

Em um texto publicado em 2018, Olivier Faure e Hervé Guillemain convidam-nos a pensar sobre a natureza política das medicinas heterodoxas. Embora o conceito se refira a uma “nebulosa” de diferentes práticas, estas possuem características em comum e uma dinâmica de trocas e circulação que as conecta entre si. Outra característica dessas medicinas é o fato de atraírem, de modo frequente, adeptos de um mesmo meio ideológico (Faure, Guillemain, 2018, p.13). Esses dois historiadores apontam para o fato de que há poucos estudos que explorem as relações entre essas “outras medicinas” e o desenvolvimento de movimentos sociais e ecologistas contemporâneos.

Essa foi a motivação que nos levou a investigar a Luta do Larzac, um movimento social ocorrido na França entre 1971 e 1981 e que hoje tem sua memória conectada à luta fundiária e ao fato de seu território abrigar a convergência de diferentes lutas. A fim de realizar esta pesquisa – circunscrita ao contexto de um pós-doutorado realizado na Universidade de Montpellier –, vários arquivos e fontes foram interrogados: de arquivos municipais, como o Arquivo de Millau, a coleções associativas, como o arquivo da Sociedade Civil das Terras do Larzac, armazenado em Montredon, e o arquivo do Cun, que estava então abrigado no vilarejo de Potensac. O levantamento resultou na coleta de uma grande quantidade de fontes ainda não utilizadas, sendo que o acesso a elas só foi possível pela ajuda incansável de diferentes militantes que habitam a região.

Para a confecção deste texto, utilizamos principalmente os documentos do Arquivo Municipal de Millau. Organizados por fundos de doação realizados na maior parte por militantes e ex-militantes, são compostos pelo material produzido pelos diferentes grupos formados a partir da Luta do Larzac, como atas, relatórios, correspondências, panfletos, entre outros. Também recortes de jornais locais e nacionais, reunidos em pastas e formando “coleções”, foram utilizados nesta pesquisa. Assim, buscamos analisar as fontes captando as dinâmicas sociais nelas subentendidas, considerando-as como discursos sociais que procuram articular corpo, identidade e território.

Para proceder à interpretação de fontes, nos valemos dos conceitos de Marc Augé sobre a importância do espaço para diferentes grupos sociais e a relação existente entre lugar e corpo. Augé (1992, p.60) afirma que o espaço tem importante papel na construção do sentido e da identidade de uma comunidade que tende a defendê-lo contra ameaças internas e externas. Citando Louis Marin, considera que o lugar é o espaço no qual o corpo está localizado (Augé, 1992, p.70), e essa relação entre um lugar – que é coletivo – e o corpo – o espaço individual – pode ser percebida de diferentes maneiras, já que é a partir da experiência própria ao corpo que as construções sobre o espaço podem ser percebidas (p.80).

A relação corpo-lugar foi marcante em termos de interpretação de saúde e doença em diferentes momentos da história. Olivier Faure ressalta essa dimensão quando analisa as analogias estabelecidas pelo homeopata Léo Simon entre o corpo e a cidade no século XIX e em movimentos sociais como o saint-simonismo e o fourierismo, nos quais a homeopatia era bastante presente, apontando igualmente as conexões entre medicinas heterodoxas e movimentos contestatórios (Faure, 2018, p.43).

Neste texto, buscamos compreender como essas duas dimensões – a luta pela preservação de um espaço, característica da Luta do Larzac, e a proteção do corpo diante de ameaças como as doenças – estavam interligadas. De modo mais direto, procuramos investigar como a Luta do Larzac abrigou debates sobre a preservação desse “espaço individual” que é o corpo pelos cuidados que o conectavam com o “lugar” – um espaço oposto ao não lugar, onde o ser humano permanece anônimo (Augé, 1992) – por meio da aproximação entre “sensibilidade ecológica” (Martin, 2023) e medicinas heterodoxas.

O local é igualmente marcado pela presença do Exército, que tem um campo militar na pequena cidade de La Cavalerie desde 1902 (Terral, 2017, p.18), servindo de campo de concentração para argelinos suspeitos de integrar a Frente de Libertação Nacional durante a Guerra da Argélia (1954-1962). O campo militar integra o Parque Natural Regional de Grands Causses, criado pelo Estado em 1995 (Terral, Späni, 2015). Neste texto, abordamos um evento específico – o Estágio de Creissels – promovido por Larzac-Université em 1976 e que permite ver alguns indícios da relação entre ambas as esferas de interesse citadas. Antes de adentrarmos nessa análise, é importante entender o que foi a Luta do Larzac, evento-chave na história dos movimentos sociais na França.

## A Luta do Larzac: primeiras mobilizações

A Luta do Larzac foi um movimento social ocorrido entre 1971 e 1981 no Sul da França e que tem uma longa história de relacionamento entre seres humanos, as ovelhas de raça Lacaune e os demais elementos que compõem esse meio. A atividade econômica principal da região consiste na criação de ovelhas, principalmente para a fabricação de laticínios como o queijo Roquefort, o que ocorre a partir do século XIX (Artières, 2021, p.24).

Sua geografia se caracteriza por um planalto calcário com 800 metros de altitude, clima seco com vegetação esparsa, sendo que sua aparência desértica foi realçada pelo êxodo rural ocorrido em toda a França durante o século XX. A mobilização em torno da conservação de terras do Larzac fez parte de um período de efervescência mundial, traduzida por contestações que englobavam as manifestações contra a Guerra do Vietnã, a emergência dos movimentos negros, da segunda onda do feminismo e de movimentos juvenis de contestação (Ferraz, 2021, p.212). Na França, os anos 1970 apresentam duas balizas temporais: os acontecimentos de Maio de 68 e a eleição de François Mitterrand em maio de 1981 (Mathieu, 2010, p.14).

Essa década é conhecida como um momento-chave na contestação de instituições, de relações sociais baseadas em autoridades e do modo de vida estabelecido no pós-Segunda Guerra e foi marcada por múltiplas dinâmicas: no caso do Larzac, ela contribui para exacerbar as tensões entre centro (Estado) e periferias (regiões), entre o mundo rural e as cidades e também a contestação ao Exército e a tudo o que ele representava, além de canalizar a vontade de experimentar outros modos de viver e se organizar (Mathieu, 2010, p.51-59).

Ao mesmo tempo, o período foi testemunha de muitos movimentos de reivindicação e de crescente politização, que alimentavam greves trabalhistas lideradas pela Confederação Francesa Democrática do Trabalho e pela Confederação Geral do Trabalho, mostrando a crescente força dos sindicatos na França (Mathieu, 2010). Entre as diferentes formas de luta presentes na miríade que compõe a década de 1970, os combates travados a partir do território e de sua defesa são os mais significativos para as reflexões acerca da Luta do Larzac. Os movimentos regionalistas estavam entre eles, assim como mobilizações antinucleares e movimentos ecológicos, compondo os “novos movimentos sociais” (Touraine, 1973).

A Luta do Larzac se iniciou quando, em 12 de outubro de 1970, o então secretário de Estado da Defesa Nacional, André Fanton, anunciou o projeto de extensão do campo militar. A notícia foi confirmada um ano depois, em rede nacional de televisão, pelo ministro de Estado responsável pela Defesa Nacional, Michel Debré (Terral, 2011, p.45). A iniciativa planejava desapropriar os camponeses que tinham suas terras dentro do perímetro de extensão do campo militar, o que gerou o movimento de resistência conhecido como Luta do Larzac, que teve uma duração de dez anos.

O início do movimento foi marcado por um evento que definiu os contornos dos anos de resistência que se seguiram: o juramento dos 103. Esse ato aconteceu após o convite de Lanza del Vasto, líder fundador da comunidade de L’Arche, localizada próxima ao Larzac, para realizar um jejum de 15 dias como forma de manifestação não violenta. Após esse ato, 103 dos 107 líderes das explorações agrícolas atingidas pelo projeto de desapropriação assinaram um juramento de não vender suas terras para a extensão do campo militar (Burguière, 2012, p.61-68).

Várias ações e mobilizações ocorreram, então, atraindo militantes de toda a França e do exterior. A luta primordial esteve centrada na reivindicação camponesa de defesa de um território que permitisse garantir a vocação agrícola da região. Embora o juramento dos 103 possa indicar a ideia de unidade do movimento, é importante destacar que houve várias críticas, principalmente de habitantes da cidade de La Cavalerie que viam na presença do Exército uma oportunidade de desenvolvimento econômico. Entre os grupos contrários à Luta do Larzac, destacou-se o “Movimento pela paz e a ordem no Larzac”, que denunciava a repressão sofrida pelos camponeses favoráveis à venda e a presença de militantes vindos de diferentes movimentos que, segundo eles, impediam o bom diálogo entre Exército e aqueles que estavam dispostos a negociar (Terral, 2011).

A importância da dimensão territorial da Luta do Larzac pode ser percebida pela historiografia que analisa o tema. Seu principal historiador, Pierre-Marie Terral, dedicou-se a coletar e analisar uma extensa tipologia de fontes, traçando os passos do movimento, as suas raízes e conexões políticas, a diversidade de ações e os desdobramentos após seu término (Terral, 2011, 2017). Terral caracteriza o movimento em diferentes fases: o período entre 1970 e 1974 corresponde ao início de um movimento de caráter local que toma uma dimensão nacional; o período entre 1974 e 1977 marca um tempo de incerteza seguido pela mobilização dos militantes em grandes manifestações e criação de grupos e estruturas de sedimentação do movimento. Por fim, o período entre 1977 e 1981 foi de união da luta local às lutas sociais e ecológicas originadas em outras regiões francesas, finalizado com a eleição de François Mitterrand e o abandono do projeto de extensão do campo militar.

Esse historiador também aponta a dificuldade em precisar o número de militantes que se engajaram na Luta do Larzac, mas ele indica que, em momentos de grandes manifestações, o local recebia entre cinquenta mil e cem mil pessoas, de acordo com as estimativas do movimento e das autoridades policiais (Terral, 2011, p.18). A análise proposta neste texto irá focar nos anos iniciais de Larzac-Université, grupo criado em 1975, e, mais especificamente, no Estágio de Creissels, ocorrido em 1976.

### A criação de Larzac-Université como espaço de resistência

Os anos seguintes ao juramento dos 103 se caracterizaram pelo que Terral chamou de “ancoragem reforçada no terreno” (Terral, 2011, p.84), manobra marcada pela criação de instituições que favoreciam a ocupação do território por atividades e grupos que o tornassem vivo. Nesse sentido, a Associação para a Promoção da Agricultura no Larzac foi criada em janeiro de 1973, e as reuniões construídas a partir dela decidiram dividir a zona de interesse de desapropriação em seis porções e instituir comissões especializadas de atuação: ação, propaganda, festas, obras, jurídico, imprensa, finanças e Grupo Fundiário Agrícola. Este último grupo surgiu como mecanismo de resistência às aquisições de terras pelo Exército, mantendo-as nas mãos do movimento e sob gestão coletiva.

Em fins de 1973, a construção do estábulo da Blaquière (Terral, 2011, p.86) deu início a uma série de obras de edificação e revitalização de pequenas comunidades no território do Larzac, como foi o caso de Truels, ocupada em 1974, do Cun, cuja primeira sede data de 1975, e de Montredon, sede de Larzac-Université, criada em 19 de maio de 1975 e instalada em 1976 (p.97), marcando a presença de intelectuais no movimento.

Em 5 de julho de 1975, Jean-Pierre Coulon, camponês e conselheiro-geral do departamento, e Jean-Marie Aubery realizaram uma visita à Universidade Paris VII, para a criação de Larzac-Université. Naquele momento, o conselho de administração da associação, composto por cerca de trinta pessoas, informou a intenção de desenvolver “ações culturais e ações de informação que interessam aos agricultores e criadores da região, mas, igualmente, a todas as pessoas interessadas por essas questões” (Gardarem..., 5 jul. 1975, p.5).^1^

No dia seguinte à criação de Larzac-Université, o periódico *Midi Libre* noticiava o evento definindo que o objetivo da associação era o de ser um espaço de inclusão e partilha de saberes: “Em Montredon, se desenrolaram estágios ou seminários abertos a todos sobre temas bem precisos, livremente propostos, organizados após a apreciação do conselho de administração ‘para mostrar que as escolas não devem ter o monopólio do ensino’” (Larzac-Université..., 20 maio 1975, p.3; destaques nossos).

Ao longo de seu primeiro ano de existência, Larzac-Université organizou vários estágios sob a responsabilidade de acadêmicos bastante envolvidos com a associação: Michel Fontaine, professor na escola de veterinária de Lyon, fez uma apresentação sobre a economia rural e as técnicas de criação animal; Marthe Gauthier, pesquisadora no Instituto Nacional de Saúde e de Pesquisa Médica, abordou a questão da energia nuclear; e o médico Alexandre Minkowski, professor de neonatologia, lançou uma série de reflexões sobre a medicina rural (Gardarem..., 5 jul. 1975).

Outros estágios foram também previstos para setembro de 1975: um sobre a energia solar, com projeto de instalação de um aquecimento solar, e outro sobre a proteção da natureza, em ressonância com a criação de dois espaços pela Sociedade Francesa de Proteção da Natureza (De la formation..., 23 maio 1975). Essa programação foi apresentada com detalhes em um relatório da assembleia de 4 de outubro de 1975 que também evocou a realização de um estágio sobre a energia nuclear e outro sobre “a energia nova”, com um estudo sobre energia solar e a construção de um aquecedor solar. O documento resultante da mesma assembleia menciona também um estágio sobre a célula, organizado por Marthe Gauthier (Larzac-Université, 4-5 out. 1975).

O programa de estágios concebido para 1975-1976 foi definido nessa mesma assembleia, que teve continuidade no dia seguinte na comunidade do Cun. Os responsáveis pelas atividades foram elencados: economia rural, com o geógrafo Raymond Gugliemo e a participação de um grupo de teatro; a continuação do estágio sobre alimentação, saúde e genética, com Marthe Gauthier; a organização de um estágio sobre outras formas de energia, por José de Félice, com destaque para a utilização de gás de estrume e o projeto de instalação de aquecedores solares; um estágio sobre as escolas rurais sob responsabilidade de um “professor escandinavo”; um estágio sobre a medicina rural proposto por Janine Raulin, pesquisadora do Centro Nacional da Pesquisa Científica, com “a participação de um correspondente local” e um convite ao doutor Minkowski para “falar sobre uma nova medicina”; um estágio sobre as escolas Freinet, a cargo de um grupo Freinet; e um estágio em história, proposto por Jean Chesneaux, da Universidade Paris VII, sobre a história do Larzac, de Millau e da região. Foi igualmente proposto um fórum intitulado “Liberdades”, sob a responsabilidade da filósofa Jeannette Colombel, questionando as relações entre Estado e instituições (Larzac-Université, 4-5 out. 1975).

A dinâmica dos membros de Larzac-Université em torno das sugestões de estágios mostra o quanto pesquisadores e universitários estavam engajados no trabalho de difusão de saberes. Nem todas as propostas foram realizadas e outras foram acrescentadas mais tarde, mas a assembleia geral de outubro de 1975 nos permite ter uma noção da diversidade de assuntos e de profissionais mobilizados, um rico universo principalmente se considerarmos que se trata do início da associação. Entre as formações organizadas, uma em particular se destaca por articular os temas meio ambiente e saúde: trata-se do Estágio de Creissels, realizado entre 30 de agosto e 2 de setembro de 1976.

### Um inusitado estágio em Creissels

Mesmo se a saúde ovina aparece como um tema privilegiado de Larzac-Université em diversas ocasiões, como na formação do grupo de veterinários em 1975 (Robinet, 2011, p.113), a saúde humana também foi um tema presente nas propostas de cursos apresentadas pela associação. Esse interesse se manifestou inicialmente nos já citados estágios sobre medicina rural e fisiologia pré-natal organizados pelo doutor Minkowski e nos estágios sobre célula e genética propostos por Marthe Gauthier.

Na assembleia de 5 de outubro de 1975, as abordagens sobre saúde, alimentação e “a ideia de uma discussão sobre a vacinação” (Larzac-Université, 4-5 out. 1975) tomam forma no estágio “Os produtos químicos, os medicamentos, a energia: uma ameaça à vida cotidiana?” realizado em Creissels, pequena cidade vizinha a Millau, no ano seguinte. O material propositivo desse estágio indicava a reflexão sobre se, “em nossa sociedade, a ciência não é utilizada para sustentar o sistema capitalista e não permite camuflar, em problemas pretensamente técnicos, as escolhas políticas que moldam nossa vida?”. Sabemos, por esse documento, que o evento ficaria a cargo de “cientistas de Grenoble” e que seus responsáveis eram Simone e Bernard Verdie – este último político da região engajado no mundo rural (Un stage..., s.d.).

O estágio aconteceu em quatro sessões diárias, segundo o programa: o dia 30 de agosto foi dedicado ao debate sobre “vacinas e medicamentos”; o dia 31, à “poluição: o Tarn, rio ou esgoto?”; o dia 1 de setembro abordou a “energia”; e no dia 2 de setembro a sessão teve como tema “adubos: cultura química, cultura biológica”. O relatório do estágio não permite uma identificação clara dos palestrantes e contém vagas indicações sobre o responsável pela primeira sessão, que “foi centrada nas questões colocadas pelos participantes sobre as vacinas e os medicamentos e nas respostas propostas por um médico em exercício em Millau” (Larzac-Université, 1976). Nessa sessão, dedicada às vacinas e aos medicamentos, o palestrante foi convidado a tratar de cinco questões: “A vacinação obrigatória é justificada, tendo em conta os riscos que ela comporta? Os medicamentos são sempre necessários para curar? As 20 mil especialidades existentes em farmácia são úteis? Não poderíamos diminuir esse número? O que pensar dos curandeiros, homeopatas, acupunturistas?” (Larzac-Université, 1976).

De acordo com o relatório do estágio, a sessão “saúde” não convenceu o público, que teceu críticas sobre o “amálgama feito pelo médico entre curandeiros, homeopatas, acupunturistas como charlatães” (Larzac-Université, 1976). Do mesmo modo, as respostas dadas pelo médico palestrante dessa sessão não satisfizeram os participantes, que julgaram que a relação entre o médico e o público reproduziu “a situação da medicina tradicional onde cada um confia a decisão sobre sua saúde completamente ao médico e ao sistema hospitalar” (Larzac-Université, 1976). Uma parte do público lamentou ter perdido a oportunidade de discutir “a questão da responsabilidade de cada um a respeito de seu corpo, sua saúde e suas doenças” (Larzac-Université, 1976).

A sessão seguinte sobre poluição se baseou no exemplo concreto do Tarn, rio que atravessa Millau, antes de ampliar a reflexão para as diferentes formas de contaminação da água por radioatividade, detergentes, pesticidas e adubos, sal das rodovias, rejeitos industriais e pelos curtumes que se desenvolveram a partir do século XIX. Em relação à energia, após ter alertado a respeito das ameaças relacionadas às reservas de petróleo e de gás natural, bem como sobre os perigosos reatores nucleares, o responsável pela sessão denunciava que “a sociedade do ‘tudo nuclear’, ‘tudo elétrico’ será mais centralizada e policialesca que a atual tecnocracia” (Larzac-Université, 1976). Às “antigas” fontes de energia se opunham as energias “novas” ou “suaves”: geotermia, energia oriunda do mar, eólica e solar. No final da sessão, “o debate permitiu precisar os perigos da radioatividade para os organismos vivos e trazer ao homem o problema da energia, pois nenhuma energia utilizável na natureza não o é sem o esforço dos homens” (Larzac-Université, 1976).

A energia nuclear, centro de críticas amplas na sociedade francesa dos anos 1970, também era vista com desconfiança pelos membros de Larzac-Université que estimulavam as experiências com as “novas energias”. Um documento anexo ao relatório da sessão dedicada à poluição apresentava os efeitos das radiações sobre o corpo humano, indicando várias vias de contaminação, distúrbios e doenças associadas à energia nuclear. Tal atenção aos efeitos da radiação se inscrevia em um olhar que percebia corpo humano e meio ambiente como elementos interdependentes e que compreendia a contaminação por meio da circulação de substâncias nocivas entre os organismos vivos: “A água de irrigação radioativa contamina as plantas que crescem em um solo irrigado, e a concentração de elementos perigosos na planta podem ser muito mais elevados do que a da água. Essa concentração pode aumentar ainda nos animais que comem esta planta e ainda mais no homem que come estes animais [*sic*]” (Larzac-Université, 1976).

Os temas das sessões seguintes prolongavam o debate sobre a relação entre humanos e não humanos por meio da alimentação, questionando principalmente os adubos e opondo “cultivo químico” e “cultivo orgânico”, pesticidas e aditivos alimentícios. Após ter apresentado os elementos necessários ao crescimento das plantas, a sessão sobre os adubos propunha a comparação entre a agricultura “de antigamente”, quando “uma comunidade familiar ou do vilarejo vivia em circuito fechado”, e a agricultura “industrial química”, que “implica o uso maciço de produtos industriais”, e “assegura, assim, o lucro das grandes sociedades multinacionais”, sujeitando “o agricultor à lógica capitalista” (Larzac-Université, 1976).

Nesse contexto, a agricultura orgânica era apresentada como “mais saudável” para os consumidores, embora a questão de sua viabilidade financeira para o agricultor fosse levantada. A inquietação relacionada ao acompanhamento do agricultor engajado no modelo de produção orgânica reapareceu na sessão sobre “pesticidas”, que, sem negar os efeitos deletérios dessas substâncias cuja aplicação seria “encorajada pela indústria química”, recomendava somente a diminuição de seu uso. Assim, a superioridade da agricultura orgânica era ressaltada pelo redator do relatório: “A agricultura orgânica considera que o solo é um elemento vivo que é necessário tratar com precaução, de modo que cultivos e animais rústicos possam nele se desenvolver naturalmente, sem serem forçados, protegidos dos inúmeros produtos químicos industrializados (adubos químicos, pesticidas, hormônios, medicamentos etc.)” (Larzac-Université, 1976).

A conclusão da crítica tecida aos “produtos químicos” concentrava-se nos aditivos alimentares, abordados na última sessão do Estágio de Creissels. Mesmo que possibilitassem o surgimento de “novos produtos alimentícios”, isso ocorria tendo como custo o aparecimento de numerosas doenças: “Alergias, cânceres, desregulações intestinais, dermatite, acidentes vasculares, cálculos renais” (Larzac-Université, 1976).

De acordo com o balanço sobre as sessões do Estágio de Creissels, houve a participação de dezoito indivíduos, sendo o público composto por camponeses, habitantes da região de Millau e pessoas vindas de Grenoble. A sessão “saúde” foi pouco apreciada e qualificada de “não interessante” por dois participantes, sendo que um deles assinalou seu desagrado como oriundo “do médico que não soube controlar e canalizar sua personalidade”. Nesse mesmo balanço, entre as manifestações por novas formações, o tema “saúde” apareceu como prioritário: “a saúde de homens e animais, a vacinação”, ao lado de “agricultura orgânica” e “informações ecológicas” (Larzac-Université, 1976), indicando que a rejeição manifestada pelos participantes não tinha relação com o tema, mas com a forma como este havia sido tratado.

Durante os cinco anos que se seguiram ao Estágio de Creissels, vários outros estágios, diversas conferências e exposições foram organizadas sobre temas próximos ao meio ambiente, à agricultura orgânica e às “novas energias”: um estágio sobre botânica, energias novas, energia nuclear, ecologia e trabalho em 1977; o acolhimento às “aulas verdes”; um estágio de reconhecimento de aves de rapina no contexto de “proteção à natureza” em 1980 (Larzac-Université, jun. 1980, p.3); a brochura “passeios no Larzac”, exposições e cofundação do Centro de Aplicação e de Promoção de Novas Energias Ecológicas em Montpellier, encarregado de desenvolver a agricultura orgânica em 1978 (Larzac-Université, set. 1979); um estágio anunciado no verão de 1978 sobre “o local da ecologia na luta dos trabalhadores: relações entre ecologia e organização do território, a saúde, a alimentação, a moradia ..., ecologia e militarização da sociedade, problemas de uma coordenação, pelos trabalhadores, de suas lutas ecológicas” (Deux..., 23 ago. 1978, p.11); grupos de estudo sobre a utilização de “métodos naturais e produzir energia” em 1979 (Une source..., 28 set. 1979) e em 1980 (Larzac-Université, 18 jan. 1980, p.2); e uma conferência sobre “o nuclear, por que e como dispensá-lo” (Larzac-Université..., fev. 1980, p.2), entre outras ações.

Em 1981, Larzac-Université propôs um estágio sobre a agricultura orgânica em 14 e 15 de março. Organizado por Nature et Progrès, ele foi realizado por um veterinário homeopata, M. Brun (Larzac-Université..., 14 mar. 1981). Um mês antes, Larzac-Université promovia uma conferência sobre a homeopatia em Millau, retomando os questionamentos presentes no Estágio de Creissels em 1976:

Larzac-Université: a homeopatia

Larzac-Université propõe uma primeira noite de informação sobre a homeopatia, uma medicina velha de mais de 185 anos e, porém, mal conhecida. Então, o que é a homeopatia? A gente a confunde frequentemente com a medicina das plantas. É completamente outra coisa: um modo de escapar à supermedicalização, de mudar as relações entre médico e paciente, onde este último tem a oportunidade de assumir o controle e compreender seus próprios males. Vocês poderão colocar suas múltiplas questões, um médico homeopata responderá a elas (Larzac-Université..., 8 fev. 1981, p.5).

Não há outros indícios sobre esse evento dedicado à homeopatia. Algumas semanas depois, a assembleia geral de Larzac-Université anunciava: “Apesar dos diálogos positivos e interessantes, o balanço financeiro negativo nos obriga a cessar temporariamente este ciclo (de conferências)” (Larzac-Université, fev. 1981, p.3). Ao final do ano de 1981, uma nova assembleia decidia ser “necessário precisar e desenvolver seus objetivos e suas atividades e, eventualmente, mudar de nome” (L’assemblée..., dez. 1981, p.6). Em 1982, a criação do Centro de Iniciativas Rurais no local de Larzac-Université confirmou essa orientação.

As relações entre saúde individual e saúde global, feitas por intermédio de diálogos entre ecologia e saúde, não se limitaram ao Estágio de Creissels ou às palestras promovidas por Larzac-Université. Simultaneamente ao seu surgimento foi criado o Cun, grupo cujo objetivo era estimular o debate e as ações pautados na não violência e dar suporte ao trabalho dos objetores de consciência.

Analisando os estágios e as formações promovidos por esse grupo, percebemos o crescente interesse por temas que perpassavam ecologia e medicinas heterodoxas: a partir de 1981, os estágios e as formações oferecidos e relacionados ao tema “saúde” privilegiaram abordagens não centradas na biomedicina. A formação “Saúde: como reforçar os mecanismos de defesa do organismo?”, por exemplo, era assim apresentada:

O interesse pelas medicinas chamadas ‘suaves’ provém de uma reviravolta na atitude diante da doença. Pois o corpo, seu meio e também seus intermediários (agricultura, alimentação etc.) devem ser considerados como elementos de um mesmo conjunto. Assim, a doença é mais frequentemente devida a um desequilíbrio interno dos mecanismos naturais de defesa que a uma agressão externa. Como reforçar esses mecanismos? (Le Cun du Larzac, 1981).

Essa formação do Cun, centrada em medicinas alternativas, ocorreu entre 1981 e 1986, continuando sob outras denominações até o final da década de 1980. Esses diferentes eventos refletiram a emergência de novas formas de articulação entre corpo, identidade coletiva e território, constituindo marcos históricos que esclarecem a aparição de um novo tipo de sensibilidade. Uma vez que a relação entre saúde e meio ambiente, com atenção ao papel das medicinas alternativas, torna-se mais visível a partir dessa década, é possível perceber o Estágio de Creissels como um momento crucial de um contexto em transformação.

## Do local ao global

Quais relações são possíveis entre saúde e meio ambiente no contexto francês dos anos 1970? É possível inscrever o interesse demonstrado pelas formações e pelos estágios promovidos por Larzac-Université nesse quadro mais amplo?

A partir dos anos 1960, com a crescente urbanização da França e sua entrada na sociedade de consumo, ocorreram mudanças na relação entre seres humanos e meio ambiente, processo que não foi exclusivo daquele país (Souza, Franco, Drummond, 2024). O período foi marcado pela lei de 22 de julho de 1960 de criação dos parques nacionais e, em 1962, pela “Delegação de organização do território e da ação regional”, responsável por orientar e implementar as políticas de organização dos territórios. Foi necessário aguardar até 1971 para que o governo do primeiro-ministro Chaban-Delmas indicasse, pela primeira vez, um ministro responsável pela proteção da natureza e do meio ambiente na pessoa de Robert Poujade. O surgimento, no seio do Estado, da preocupação com o equilíbrio do território e a proteção do meio ambiente correspondeu ao nascimento da ecologia na França (Vrignon, 2017, p.215).

Embora a pauta ecológica tenha tomado uma dimensão política mais precisa com a candidatura de René Dumont à presidência da República, em 1974, vários grupos que defendiam essa agenda tomaram forma desde o final da década anterior, como a Federação Francesa de Sociedades de Proteção da Natureza, criada em 1968, e os Les Amis de la Terre, em 1970. Dentro dessa esfera, os movimentos antinucleares foram centrais, sendo impulsionados pela crise do petróleo de 1974, que estimulou a construção de centrais nucleares como alternativa energética. As duas principais lutas antinucleares na França aconteceram em Creys-Malville (1977) e Plogoff (1978) (Mathieu, 2010). Esses movimentos tiveram eco no Larzac, como indicam diferentes fontes coletadas.

A fim de compreender a influência da sensibilidade ecológica no interesse por práticas alternativas em saúde, é preciso destacar dois pontos complementares. O primeiro diz respeito ao fato de a Luta do Larzac estar centrada em um objetivo bem preciso, determinado pelos camponeses a partir do juramento dos 103: garantir que a desapropriação das terras intencionada pelo governo francês não fosse efetivada, reafirmando a vocação agrícola do território. Na década de 1970, os agricultores não tinham como meta a mudança na forma de produção visando a uma agricultura orgânica: a tendência do mundo rural em se preocupar com a qualidade da alimentação em conjunto com a preservação ambiental se desenvolveu em maior escala entre 1980 e 1990 (Martin, 2023, p.14).

O segundo ponto diz respeito ao fato de que a atenção do mundo agrícola antes da década de 1980 estava voltada muito mais para a modernização da agricultura, para a adequação desta ao capitalismo industrial e ao êxodo rural. Embora esse cenário estivesse intimamente ligado à forma de produção criticada pelos ecologistas, a questão maior se centrava nas condições de vida e de trabalho dos agricultores. A “esquerda camponesa” inicialmente criticava a perda do controle da produção e temia que o interesse por “outras práticas agrícolas” pudesse desviar as lutas consideradas prioritárias. Esse contexto – presente na Luta do Larzac – transformou-se progressivamente ao longo da década de 1970, quando se intensificaram a circulação, no mundo rural, de pessoas e ideias com perfis sociais bastante diversos. No caso do Larzac, isso significou a conexão de uma luta local a movimentos nacionais e globais.

Apesar dos anos 1970 e 1980 serem marcados pela emergência da ecologia política na França, é preciso considerar que denúncias a respeito dos malefícios da industrialização e da urbanização sobre a saúde ocorriam desde o século XIX na Europa, onde a “natureza” era concebida como sendo regida por leis que deveriam ser obedecidas sob pena de romper com o equilíbrio necessário à manutenção da saúde humana. Nesse contexto, o modo de vida moderno aparecia como destruidor das capacidades de resistência do organismo; ao contrário, uma higiene de vida bem estabelecida por uma alimentação saudável, exercícios físicos e banhos de sol contribuiria para restaurar esse equilíbrio (Vrignon, 2017, p.60-61).

Nos anos 1960 e 1970, os ecologistas se reapropiaram dessas considerações em um novo contexto: a relação entre meio ambiente e saúde, entre território e ser humano foi o centro de interesse de certos pesquisadores franceses – como Roger Heim, diretor do Museu de História Natural de 1951 a 1965 e um dos fundadores da União Internacional pela Proteção da Natureza –, que também se relacionavam com comunidades transnacionais. Eles denunciavam a transformação caótica dos espaços, considerada nociva para os equilíbrios naturais e, por consequência, para a existência humana. A saúde aparecia como uma condição diretamente ameaçada pela difusão de produtos químicos e radioativos ao longo da cadeia alimentar, na qual o ser humano figurava como seu último elemento (Vrignon, 2017, p.39-40).

A confluência entre meio ambiente e saúde também participou da ascensão das medicinas alternativas ou heterodoxas na França nos anos 1970. Vale a pena ressaltar que existe uma apaixonante discussão sobre como nomear as práticas em saúde que utilizam paradigmas diferentes da biomedicina. O historiador Olivier Faure, por exemplo, insiste no fenômeno de emergência dessas medicinas no século XIX como altamente conectado às mudanças ocorridas na biomedicina: assim, ele prefere denominá-las “métodos médicos marginais” (Faure, 2015, p.319) ou, mais recentemente, “medicinas heterodoxas” (Faure, Guillemain, 2018, p.13), seguindo a proposição de Roberta Bivins (2007) de que essas medicinas se caracterizariam pelo contraste à ortodoxia da biomedicina.

De qualquer forma, Faure e Guillemain afirmam que, embora façam parte de escolas bem distintas, essas medicinas formam uma nebulosa na qual os intercâmbios de conceitos e práticas as caracterizam, ideia igualmente partilhada por Laurence Monnais (2016, p.213), que reivindica uma “permeabilidade dos sistemas”, principalmente no nível terapêutico. Outros pontos em comum a essas medicinas são o fato de que elas fazem parte de um *cultic milieu*, arregimentando adeptos de um mesmo meio ideológico e que associam mais de uma medicina em suas possibilidades terapêuticas (Faure, Guillemain, 2018, p.13).

Como “medicinas dentro da medicina”, as medicinas heterodoxas surgem na Europa nos séculos XVIII e XIX e se desenvolvem propondo soluções às questões ligadas à profissão médica. A medicina naturista – uma das formas alternativas à biomedicina – conheceu sua inserção em diferentes partes do mundo (Braga, 2018) a partir da experiência europeia. Na França, na segunda metade do século XVIII, ela opôs os adeptos do intervencionismo médico àqueles que aconselhavam a prudência e a observação diante de um quadro patológico. Essa medicina, pautada na ideia de natureza curadora (*natura medicatrix*), também estava baseada no vitalismo, isto é, na ideia de um princípio vital que animava o organismo, defendendo a importância de se observar o curso da doença e permitir o reequilíbrio “natural”, em vez de intervir com soluções medicamentosas, respeitando sempre o princípio da individualização de cada doente (Villaret, 2018, p.85).

Nos anos 1820 a 1840, a homeopatia, a acupuntura e o magnetismo tentaram propor novas maneiras de curar na França e, no período entreguerras, ofereciam um ponto de vista diferenciado em relação à saúde, considerando o “terreno” mais importante do que a doença, observando a singularidade de cada organismo na resposta aos quadros patológicos (Faure, 2015, p.321). Em sintonia com esse movimento, em 1926 o termo “holismo” foi criado, defendendo a ideia de que o todo integral não era apenas a soma de todas as partes, mas a relação entre vários fatores, incluindo sociais e psicológicos, conceito que inspirou as medicinas heterodoxas a partir de então (Monnais, 2016, p.215).

Algumas terapêuticas marginais se aliaram a movimentos sociais, uma vez que seus adeptos acreditavam que mudar a medicina também poderia ser uma via de mudança social. Nesse sentido, no século XIX tivemos médicos homeopatas também engajados no saint-simonismo, como Léon Simon, e no fourierismo, como Fleury Imbert (Faure, 2015, p.326) ou como Benoît Mure no Brasil.

Após a Segunda Guerra Mundial, mais precisamente nas décadas de 1960 e 1970, as reivindicações por mudanças nas ofertas em saúde foram nutridas por movimentos sociais – como a Contracultura e o Maio de 68 – e também pelo crescente individualismo, que demandava maior poder de escolha e decisão inclusive sobre o corpo e a saúde. Esse “retorno” de práticas alternativas em saúde representou uma resistência ao modelo epistemológico da biomedicina, no qual especializações, dependência da tecnologia e autoridade incontestada do médico construíram uma extrema fragmentação do corpo e no qual o indivíduo cedia seu lugar central à doença na relação entre médico e “paciente” (Monnais, 2016, p.216).

O combate à iatrogenia foi, igualmente, um elemento a ser considerado na atração exercida pelas medicinas heterodoxas. Medicamentos de síntese e vacinas foram elementos do arsenal terapêutico muitas vezes recusados pelos adeptos das “outras medicinas”. No caso das vacinas, Laurence Monnais afirma não serem consideradas “medicamentos como os outros” (Monnais, 2016, p.204), podendo provocar reações de recusa, ceticismo ou hesitação.

Múltiplas foram as causas de rejeição em relação à vacinação ao longo do tempo: as más condições de estocagem, o uso de material indevido para sua inoculação e mesmo sua ineficiência em alguns casos pode ter alimentado “uma memória cumulativa sobre a toxicidade da vacina”. Além disso, existia a reivindicação do poder de decidir sobre a sua própria saúde, a crítica à obrigatoriedade imposta por sistemas de saúde que não tinham adesão ou credibilidade da população e a adesão a conceitos como o de valorização do “terreno” individual, ou seja, o corpo, como responsável pela saúde e doença, conceito defendido pelos adeptos de Béchamp, em rivalidade a Pasteur, no final do século XIX (Monnais, 2016, p.205-206).

Também a medicina naturista ou “natural” ganhou novos contornos nas décadas de 1960 e 1970 (Bernard, 30 jan. 2024). Léo Bernard indica que a renovação dessa medicina na França esteve ligada à atuação do professor de educação física Pierre-Valentin Marchesseau, por meio de suas publicações periódicas sobre o tema, e da fundação, em 1966, da Sociedade Francesa de Naturopatia, que se transformou, em 1969, na Federação Francesa de Naturopatia, Humanismo e Sociologismo Biológico.

O historiador afirma que Marchesseau pode ser considerado um dos precursores da ecologia política na França, não apenas por sua atuação intelectual, mas pela fundação do Partido Bio-Humanista, em 1972. Seu pensamento foi marcado pelo movimento ecológico dos anos 1970 e pela agricultura orgânica emergente desde a década anterior, com aproximações crescentes entre a “medicina natural” e os movimentos ecológicos (Bernard, 30 jan. 2024). Diante desse panorama geral, resta-nos compreender como e por que esses elementos estiveram presentes de forma particular na Luta do Larzac e, de forma mais específica, no Estágio de Creissels.

## Um território e múltiplas lutas

A defesa das terras dos camponeses do Larzac diante da desapropriação intencionada pelo Exército constituiu o motivo original e principal do movimento iniciado em 1971. Esse objetivo esteve no centro do juramento dos 103, que fixou o princípio segundo o qual os camponeses seriam os primeiros a dar a direção à luta. O movimento construiu, porém, convergências com outros grupos mobilizados por lutas regionais, antinucleares, ecologistas, antimilitaristas, não violentas e também as dedicadas à objeção de consciência.

Assim, em seu exercício de compreender os movimentos contestatários dos anos 1970, Mathieu considera o Larzac como um exemplo característico da impossibilidade de realizar distinções e separações muito rígidas diante das fortes conexões e imbricações das lutas que impedem uma classificação muito estrita (Mathieu, 2010, p.69).

Um dos resultados do movimento foi a constituição de uma grande rede de solidariedade e de relações de sociabilidade, permitindo debates e ações sobre a maneira de construir um espaço comum. Uma comunidade que reivindicava uma relação de continuidade com as práticas do passado, mas também de transformação, enriquecendo-se com a experiência de recém-chegados originários do meio urbano: cabe destacar a criação dos Comitês Larzac por toda a França e das associações de apoio ao movimento, cujos membros iam esporadicamente e por vezes se instalavam permanentemente no Larzac (Terral, 2011, p.52-53).

Essa convergência de desejos em torno da defesa das terras, do apoio à causa camponesa e da sua valorização não significou a ausência de tensões e conflitos entre os grupos mobilizados. Pierre-Marie Terral (2011) demonstra, ao contrário, a que ponto a chegada de pessoas externas ao núcleo inicial do movimento, constituído pelos camponeses em vias de expropriação, provocou tensões entre eles e os diferentes grupos que lá coexistiram.

Apesar de saúde e meio ambiente não serem o objetivo da luta do Larzac, o bem-viver resultante dessa equação aparecia como uma busca presente nos diversos grupos formados, inscrevendo-se em uma dinâmica mais ampla. Nela se encontram os diferentes componentes do que Vrignon (2017) designa de “nebulosa” decorrente do Maio de 68, constituída por associações e grupos como Les Amis de la Terre (1970), Nature et Progrès (1964) e Survivre (1970), que também estiveram ligados a periódicos ecologistas publicados no período, como *Survivre*, *La Gueule Ouverte* e *Écologie* ^2^ (1972) e *Le Sauvage* (1973). A essas revistas, que circulavam entre os militantes do Larzac, somavam-se periódicos como *L’Impatient* (1977), importante meio de divulgação sobre as medicinas heterodoxas e práticas alternativas na França e que foi um dos títulos constitutivos da biblioteca do Cun. Essa dinâmica existente no movimento é igualmente sustentada pelas pesquisas do historiador Jean-Philippe Martin (2023, p.32-34).

À medida que a década de 1970 avançava, a relação entre ecologia e combate ao produtivismo se fazia mais evidente na nova esquerda camponesa. A participação em movimentos contra a energia nuclear, na Luta do Larzac e em combates contra o uso de certos equipamentos agrícolas, proporcionou o encontro entre militantes ecologistas e camponeses que, progressivamente, desenvolveram o que Jean-Philippe Martin denomina “sensibilidade ecológica” (Martin, 2023, p.33). Esse processo ficou evidente na Luta do Larzac e foi mais precisamente demonstrado no Estágio de Creissels, indicativo da sistemática penetração, no Larzac, de debates e combates vindos de outras lutas.

Os militantes oriundos do meio urbano e que participaram esporadicamente dos Comitês Larzac, da instalação no território como neorrurais ou da constituição de grupos como Larzac-Université e do Cun contribuiram, igualmente, para a introdução das reflexões nutridas pelo pós-Maio de 68, pautadas na rejeição à sociedade industrial e de consumo e no desejo por um outro modo de vida, aspectos importantes tanto para o movimento ecologista quanto para os adeptos de “medicinas naturais”.

Como destacou o historiador Pierre-Marie Terral, a ecologia relacionava-se, antes de mais nada, com a receptividade do público ao assunto, mais do que por ser um ponto central do movimento. Porém, se o objetivo central era o de impedir a desapropriação de terras, a Luta do Larzac também foi percebida como uma luta “em defesa da natureza” (Terral, 2011, p.133). O território se transformou em “um lugar singular da ecologia” (Terral, 2017, p.134), reunindo militantes originários de movimentos antinucleares e de defesa do meio ambiente: a Luta do Larzac, com seu modelo de resistência não violenta ao Exército, foi inspiração para movimentos antinucleares (Vrignon, 2017, p.215).

Os debates promovidos pelo Estágio de Creissels e outros eventos de Larzac-Université representaram apenas o início da difusão de questionamentos acerca da relação entre meio ambiente e saúde no mundo rural, apontando para a interconexão entre esse debate e o interesse por “alternativas” no universo da saúde e da doença, usualmente dominado pelos conhecimentos da biomedicina. Embora o Estágio de Creissels tenha sido pioneiro ao pensar a relação entre território e indivíduo, outros eventos se seguiram a ele, principalmente na década posterior e liderados por outro grupo, conforme abordamos.

Também é importante ressaltar que as questões e críticas que emergiram na sessão “saúde” do Estágio de Creissels se conectavam a vários aspectos característicos da dinâmica coletiva do Larzac. Primeiramente, as considerações em relação a uma medicina centrada no uso de medicamentos e vacinas e a uma posição de superioridade incontestável do médico, vividas como um entrave à participação do “paciente”, revelavam a vontade, por parte dos participantes do estágio, de exercer o controle sobre seu próprio corpo e sobre os processos de saúde e doença. Ter o protagonismo sobre seu “território-corpo” parecia coincidir com a luta pelo poder de decisão sobre os destinos de seu território-espaço – o Larzac.

Uma saída para os cuidados com a saúde e que apontava para a possibilidade de uma tomada de decisão do “paciente” parece ter vindo das medicinas heterogêneas, aqui representadas pelos “curandeiros, homeopatas e acupunturistas” rotulados pelo médico palestrante de “charlatães”, de forma indistinta. Além de representarem saídas a uma terapêutica centrada no medicamento, essas “outras medicinas” acenavam com cuidados com a saúde que poderiam ser menos dependentes do Estado e de suas imposições, embora a história dessas medicinas nos mostre que elas não estiveram distantes da biomedicina preconizada pelo poder centralizado (Faure, 2018, p.29-45). A falta de interesse do profissional em responder positivamente às perguntas dos participantes do Estágio de Creissels provocou a denúncia em relação à postura autoritária do médico que reproduzia as relações já existentes em clínicas e hospitais. Afinal, em se tratando de um evento promovido por Larzac-Université, cujo objetivo era o de estimular e promover a igualdade entre saberes, o autoritarismo expresso pelo poder do médico parecia contraditório.

A dicotomia entre “artificial” e “natural” encontrada na avaliação da sessão “saúde” também ganhou espaço nas sessões sobre “poluição”, “energia” e “agricultura química x agricultura orgânica”, indicando que os questionamentos sobre as necessidades “criadas” pela sociedade – na medicina representada pelos produtos da indústria farmacêutica – expandiam-se para toda a indústria química. O interesse por uma agricultura que considerava o solo como elemento vivo ia ao encontro de uma abordagem já existente na França antes da década de 1970 e que foi retomada com maior vigor nesse período (Péssis, 2020), reforçando a correlação entre território e indivíduo, ambos considerados como seres animados. Igualmente, é possível detectar certa preocupação em relação às dificuldades financeiras que os camponeses poderiam enfrentar diante desse tipo de agricultura, reflexões próprias ao conjunto da esquerda camponesa na época (Martin, 2023).

Interpretados como inimigos, mesmo se o uso eventual fosse visto como inevitável, os adubos químicos, juntamente com os pesticidas, eram apresentados como causadores da má qualidade dos alimentos, afetando a saúde, tanto humana quanto ambiental. Essa visão mais alargada de saúde, para além da presença de doenças e seu “combate” via medicamentos, era complementada pela análise do meio, que deveria ser livre também de outros dejetos das indústrias química e nuclear. A relação com as “outras medicinas” se completava por intermédio de todo um modo de vida que deveria ser alterado para que o ser humano pudesse viver saudável e que incluía uma interdependência equilibrada com os não humanos. Esse “modo de vida natural” também era defendido pela naturopatia que, como vimos, dialogava estreitamente com os ideais ecológicos do período.

A crítica aos “produtos químicos” como causadores de doenças também provinha de uma análise global da função da ciência na sociedade francesa de então. Desde 1950, a França investia massivamente na ciência como base do crescimento econômico por meio do aperfeiçoamento tecnológico, movimento que também foi responsável por acelerar o êxodo rural e os seus desdobramentos bastante conhecidos no Larzac. O desenvolvimento de grandes programas tecnológicos nos campos nuclear, da engenharia aeroespacial e da informática também favoreciam os laboratórios farmacêuticos e a indústria química, com medicamentos e pesticidas fazendo parte de um mercado em pleno crescimento.

Embora a maioria dos cientistas se beneficiasse desses investimentos, uma minoria se insurgiu contra esses programas, denunciando o crescimento da sociedade industrial: o maior exemplo está no grupo Survivre, criado no pós-Maio de 68 pelo matemático Alexander Grothendieck (Péssis, 2014, p.7-8). No Larzac, a movimentação de pesquisadores críticos quanto aos usos da ciência também se fez presente com um encontro, ocorrido em 1981, entre cientistas e camponeses (Programme..., 1981).

Por fim, é necessário ressaltar que os grupos criados durante a luta contribuíram para reforçar o papel dos recém-instalados no Larzac – os militantes que se agregaram aos camponeses que já habitavam no local – e para potencializar a integração de outras lutas a esse movimento social. Isso favoreceu a introdução e a apropriação de novas práticas cotidianas, como foi o caso de Larzac-Université e de outras comunidades, como o Truels e o Cun. A organização de eventos sobre a recusa do nuclear, a agricultura orgânica e as medicinas “naturais” respondiam ao interesse de conhecer vias alternativas de existência, cuja busca não foi apenas teórica: ela implicou diversas ações que visavam ocupar e defender o território ameaçado e, igualmente, construir um cotidiano coerente com as crenças dos diferentes grupos formados pela luta do Larzac.

## Considerações finais

O Estágio de Creissels sinaliza um momento importante na Luta do Larzac, que se intensifica com a proximidade da década de 1980: o entrelaçamento entre uma luta local com movimentos nacionais e internacionais, especialmente com o movimento ecológico e antinuclear. Esse quadro se tornou possível com a vinda de militantes urbanos com perfis sociais bastante diversos e que estavam engajados nessas outras lutas, afetando e transformando uma luta local e conectando-a a outras lutas globais.

Esse “encontro de lutas” permitiu a entrada de ideias que anteriormente não afetavam o núcleo central de militantes do Larzac, formado pelos 103 camponeses que fizeram o juramento de não vender suas terras ao Exército, oportunizando a aproximação entre as questões territoriais e a manutenção do bem-estar do corpo individual, por meio das relações construídas em torno da saúde humana e da saúde do meio ambiente.

Embora as fontes consultadas apresentem limites – foram produzidas pelos próprios militantes e muitas vezes não exploram detalhes importantes dos eventos –, é possível, pelo cruzamento com outras fontes e com a perspectiva mais ampla de contestações que se desenrolaram no mesmo período, propor a análise de aspectos da Luta do Larzac pouco explorados e que ficaram como pano de fundo para objetivos mais imediatos do movimento, mas que foram formadores da articulação entre as noções de corpo, identidade e território.

## Data Availability

Não estão em repositório digital.

## NOTAS

1 Esta e as demais citações de obras em francês têm tradução livre.

2 A revista *Écologie* é um bom exemplo da relação entre ecologia e saúde. Tendo como redator-chefe o militante ecologista Jean-Luc Burgunder, o periódico se empenhou, na década de 1970, em denunciar o avanço das centrais nucleares e dedicou artigos sobre a Luta do Larzac. Em seus números, a revista anunciava a possibilidade de aquisição de dossiês especiais sobre “técnicas suaves: *habitat* e sociedade” ou “as vacinações não fazem regredir as epidemias” (Librairie…, 1978, p.3).
